# Bridging teacher motivation and instruction: Relevance of student‐oriented goals for teaching alongside personal achievement goals and self‐efficacy

**DOI:** 10.1111/bjep.12776

**Published:** 2025-04-17

**Authors:** Martin Daumiller, Hanna Gaspard, Oliver Dickhäuser, Markus Dresel

**Affiliations:** ^1^ Ludwig‐Maximilian‐University Munich Germany; ^2^ University of Augsburg Augsburg Germany; ^3^ Dortmund University Dortmund Germany; ^4^ University of Mannheim Mannheim Germany

**Keywords:** instruction, motivation, orientation, school, teaching

## Abstract

**Background:**

Achievement goals and self‐efficacy are key components of teacher motivation and crucial for teaching quality and student outcomes, yet the processes explaining why they lead to specific teaching behaviours remain unclear. This study focuses on student‐oriented goals as a potential process element and construct in its own right.

**Aims:**

We aim to uncover the associations of teachers' personal goals and self‐efficacy beliefs with specific teaching behaviours, and the added value of student‐oriented goals for these processes.

**Sample:**

70 secondary school teachers from German general education secondary schools, teaching Mathematics in grades 7–9 in lower track secondary education (42 women, 28 men; mean age 43.7 years, *SD =* 10.6) filled out a total of 345 lesson diaries over 5 weeks.

**Methods:**

After reporting personal goals, self‐efficacy and student‐oriented goals, teachers filled out standardized lesson diaries on their specific teaching behaviours encompassing both mastery‐based (interestingness, cognitive stimulation, individualization, autonomy support, structuring, collaboration, heterogeneous grouping) as well as performance‐based aspects (public negative feedback, homogeneous grouping and competition).

**Results:**

Two‐level path modelling indicated that personal performance goals are positively related to student‐oriented performance goals, with student‐oriented mastery goals statistically predicted by teachers' self‐efficacy. In turn, student‐oriented mastery goals positively predicted mastery‐based teaching practices. Different linkages were observed for different teaching behaviours.

**Conclusions:**

The findings highlight the relevance of considering student‐oriented goals in better understanding the relationship between teacher motivation and instructional practices.

## INTRODUCTION

It has already been established that teacher motivation matters for teaching quality and student outcomes—however, the processes linking components of teacher motivation and specific teaching behaviours are still largely unclear (Bardach & Klassen, 2021; Lazarides & Schiefele, 2021; Zee & Koomen, 2016). In the present work, we take a cognitive perspective and focus on teachers' self‐related and student‐related goals to better understand these processes. In terms of teachers' motivation, we focus on teachers' personal goals and their self‐efficacy, both of which have been postulated to influence goal‐setting processes (Daumiller, Fasching, et al., 2022; Tschannen‐Moran et al., 1998).

Following an achievement goal approach, Daumiller, Fasching, et al. (2022) introduced student‐oriented goals set by the teacher as a mediator between teachers' personal goals and their teaching behaviours. Opposed to personal goals that focus on improving own competences and the demonstration of personal competence, student‐oriented goals are goals that teachers set regarding their students (e.g. aiming at the development and evaluation of students' competence). The relevance of such goals has been highlighted in the initial work on teachers' achievement goals by Butler (2007), who emphasized that ‘teachers' goals are to a significant extent defined in terms of the achievement and well‐being of others and namely their students’ (p. 251). Daniels et al. (2013) provided a similar argument by investigating intended classroom goal structures in terms of the overall goal structures teachers intend for their classroom environment in relation to teachers' personal achievement goals.

In the present work, we follow up on such student‐oriented goals as a potential process element between teachers' personal motivation and their instructional behaviours, and expand on these processes, by (1) including, as additional predictors, self‐efficacy beliefs as key expectancy‐related aspect of motivation and work‐avoidance goals as another relevant goal besides mastery and performance goals and (2) investigating a set of diverse and specific aspects of teaching behaviour following contemporary calls to consider differences among instructional practices (see Daumiller et al., 2023). We thus integrate the two strands of research on teachers' achievement goals (e.g. Butler, 2007; Retelsdorf et al., 2010) and on teacher self‐efficacy (e.g. Klassen & Tze, 2014; Tschannen‐Moran et al., 1998) and test the relative strengths of these two motivational constructs in informing the goals they set for their students and classroom instruction. Doing so, we intend to contribute to a more nuanced perspective of relevant goal‐related processes linking teacher motivation to what teachers do in the classroom.

### Teachers' achievement goals and self‐efficacy as important aspects of teacher motivation

Understanding the motivation behind teaching behaviours is essential for improving instructional practices. Teacher motivation is pivotal in shaping not only teachers' professional experiences but also the educational outcomes for their students (Fives & Buehl, 2016; Lauermann & Butler, 2021). Previous research on teacher motivation (Daumiller, Fasching, et al., 2022; Frenzel et al., 2009; Lauermann & Butler, 2021; Lazarides & Schiefele, 2021; Zee & Koomen, 2016) suggests that teaching behaviours are a main pathway between teacher motivation and student outcomes. However, the mechanisms explaining how teacher motivation leads to specific teaching behaviours remain unclear (Zee & Koomen, 2016), calling for specific investigations thereof (Lazarides et al., 2024).

To understand teacher motivation and its effects, we must consider that schools are achievement arenas, characterized by both learning its performance affordances wherein teachers, like their students, pursue various personal and professional goals (Butler, 2007). Along with that, teachers' beliefs about their own competencies are crucial. Achievement goals and self‐efficacy beliefs are two key aspects of motivation, accordingly, that are likely to matter for teachers' approaches towards their students, their instruction and actual teaching behaviours. Taking a cognitive perspective, we focus on these constructs in the present work. Their joint consideration allows for an integration of these two theoretical strands and can provide evidence regarding their relative strength and relevance for these processes.

The achievement goal approach posits that individuals' goals in achievement contexts shape their definitions of success, task engagement, emotional experiences and learning outcomes (Daumiller, 2023; Elliot & Hulleman, 2017; Murayama & Elliot, 2019; Senko, 2016; Urdan & Kaplan, 2020). Although further distinctions and additional types of goals might also be possible and warranted, in the context of teaching, researchers typically distinguish between teachers' mastery, performance approach, performance avoidance and work‐avoidance goals (Butler, 2007; Daumiller, Fasching, et al., 2022; Papaioannou & Christodoulidis, 2007; Retelsdorf & Günther, 2011; Watt et al., 2021). These self‐directed goals go along with different views towards competence and shape teachers' cognition and behaviour.

*Learning goals* (the facet of mastery goals mainly addressed in teacher motivation research; Daumiller et al., 2019) represent a teacher's aim to develop their own competence, knowledge and skills. Teachers who pursue such goals focus on learning, improving their teaching practices and gaining a deeper understanding of their subject matter. Research has documented that such goals are associated with positive outcomes including adaptive attitudes towards help‐seeking, higher job satisfaction and ongoing professional development as well as mastery‐oriented and cognitively stimulating instruction (e.g. Butler, 2012; Butler & Shibaz, 2008, 2014; Cho & Shim, 2013; Han & Gao, 2023; Retelsdorf et al., 2010). A learning‐goal‐oriented teacher would likely interpret student help seeking as a quest for knowledge, react positively to students' questions in class and apply an individual reference norm when evaluating students' performance (Butler, 2014).

*Performance approach goals* involve a teacher's aim to demonstrate their competence relative to, and in front of, others. Teachers with performance approach goals strive to be recognized as competent and successful. The linkages of performance approach goals on teachers' behaviours and cognitions are mixed. While some studies indicate that these goals can motivate teachers to achieve high standards and improve performance, others report that they are linked to competitive and potentially stressful teaching practices, including frequent testing, grading students relative to one another, encouraging competition among students and recognizing and rewarding high achievers (e.g. Daumiller et al., 2023; Daumiller, Fasching, et al., 2022; Han & Gao, 2023).

*Performance avoidance goals* reflect a teacher's aim to avoid demonstrating a lack of competence. Teachers who adopt performance avoidance goals are often driven by a fear of failure and a desire to avoid negative judgements from others. This orientation is linked to negative outcomes including maladaptive attitudes, increased professional stress, less professional learning and higher rates of absenteeism (e.g. Daniels et al., 2013; Daumiller & Dresel, 2023; Daumiller, Janke, et al., 2022; Gorozidis & Papaioannou, 2014). Such teachers may avoid challenging tasks and innovative practices, potentially stalling their professional growth and negatively impacting their effectiveness. A performance‐avoidance‐oriented teacher might attribute students‐help‐seeking to a lack of ability and react more negatively to students' questions or requests for help while also being more likely to use a social reference norm when evaluating a student's ability or achievement (Butler, 2014).

Finally, *work avoidance goals* represent a teacher's aim to get through their tasks with minimal effort (Nicholls, 1989). This orientation is often associated with negative outcomes, including lower job satisfaction, less professional development, a higher likelihood of burnout, less engagement in professional development activities and a reluctance to adopt new teaching methods (e.g. Daumiller et al., 2021; Daumiller, Fasching, et al., 2022; Han & Gao, 2023; Nitsche et al., 2013). A work‐avoidance‐oriented teacher would likely react to student‐help‐seeking with a preference for referring them to others (e.g. other colleagues).

While achievement goals provide meaning and value to different teaching behaviours, self‐efficacy is integral for an individuals' expectancy beliefs. According to Social Cognitive Theory (Bandura, 1986, 1997), individuals strive for a sense of agency by setting goals and trying to attain them. Self‐efficacy is central for this sense of agency, as high levels of self‐efficacy enhance individuals' engagement in activities that foster learning success, such as goal setting, monitoring progress, creating effective learning environments and being persistent when things become difficult (Schunk & DiBenedetto, 2020). Applied to teaching, teacher self‐efficacy refers to teachers' beliefs in their ability to pursue their teaching‐related goals and to effectively perform teaching tasks in various situations, even under challenging circumstances (Klassen et al., 2009; Lazarides & Warner, 2020; Tschannen‐Moran et al., 1998). Tschannen‐Moran et al. (1998) proposed a model highlighting the cyclical nature of teacher efficacy, suggesting that highly self‐efficacious teachers set realistic and achievable teaching goals, invest effort in attaining these goals and persist even when facing difficulties. These teachers can analyse teaching tasks relative to their competencies, facilitating high‐quality instruction and being more resilient when faced with setbacks (Lauermann & Berger, 2021; Skaalvik & Skaalvik, 2010; Zee & Koomen, 2016). Such teaching behaviours, subsequently, are proposed to also result in higher self‐efficacy. Drawing on the model by Tschannen‐Moran et al. (1998), in the present study, we investigate the effects of teacher self‐efficacy on their student‐oriented goals and instructional practices.

### Student‐oriented goals and their relevance as a cognitive link from personal motivation to teaching behaviours

Taken together, both teachers' personal goals and self‐efficacy should influence setting student‐oriented goals, effort and persistence in teaching. While the direct impact of teacher motivation on teaching quality is well‐established, only little is known about the specific process elements. Accordingly, Lazarides et al. (2024) emphasize the critical role of investigating mediating factors, to which end they provide a model positing that teachers' motivation, including their self‐efficacy beliefs and personal achievement goals, influences their instructional practices through situational motivation and intrapersonal and behavioural processes as intermediaries. Among these are student‐oriented goals—a recently suggested cognitive driver that we focus on in the present work.

Building on an achievement goal approach, this perspective provides a lens to understand how teachers' personal goals and self‐efficacy beliefs are translated into their teaching behaviours. Specifically, regarding teachers' achievement goals, Daumiller, Fasching, et al. (2022) recently introduced student‐oriented goals, proposing that teachers' achievement goals influence their student‐oriented goals, which in turn help to shape their instructional practices. Opposed to personal, self‐directed achievement goals, which are focused on the standards and conceptions of competence that teachers hold for themselves, *student‐oriented goals* reflect the goals teachers hold for their students' learning and achievement. Informed by an achievement goal approach, these goals can be mastery‐oriented (focused on understanding and skill development) or performance‐oriented (focused on achieving better results compared to peers). While analogous to personal achievement goals, further goal types and distinctions are also plausible; this classic dichotomy was employed in a first study by Daumiller, Fasching, et al. (2022) to introduce these goals and was found to describe the data well. Student‐oriented mastery and performance goals emerged as independent constructs that were pursued by the teachers to meaningful extents. Moreover, teachers' personal mastery goals positively related to student‐oriented mastery goals (i.e. aiming for students to continue learning and improving), which in turn positively predicted students' reports of teachers' mastery practices in class. However, teachers' mastery goals were not directly linked with student‐oriented performance goals (i.e. aiming for students to score well on exams) but predicted performance practices directly and negatively (entailing public negative feedback and competition; Daumiller, Fasching, et al., 2022). Moreover, teachers' performance approach goals were positively related to student‐oriented performance goals, which then negatively predicted student reports of mastery practices in the classroom.

Teachers' student‐oriented goals are expected to be influenced by their personal goal pursuit due to two theoretical processes. First, achievement goals go along with different types of successes or competencies that teachers value most for themselves, and, by extension, shape their goals for their students and the classroom. Second, these goals may become manifest and pursued due to their functionality for achieving these more proximal self‐directed goals: For example, to achieve a personal performance approach goal (e.g. seeking to be a better teacher than others), it may be conductive to set forth student‐oriented performance goals (e.g. wanting one's students to demonstrate high performance). To this end performance practices (e.g. emphasizing competition, public comparisons and high achievement relative to peers) should ultimately also prove fruitful and be proximal to actual (in this case performance‐based) instructional practices.

Similarly, teacher self‐efficacy can be expected to matter for how teachers endorse specific student‐oriented goals: Highly self‐efficacious teachers should be more willing to adapt their instruction and assessment strategies to their students' needs rather than pressuring students to adhere to normative standards and thus, should apply mastery‐oriented practices more than performance‐focused practices (Lüftenegger & Muth, 2024; Wolters & Daugherty, 2007). Indeed, research has shown teacher self‐efficacy to be positively related to mastery‐oriented practices (e.g. Lazarides et al., 2018; Skaalvik & Skaalvik, 2023; Wolters & Daugherty, 2007). Adding to the cognitive pathway outlined before, alongside goal pursuit, self‐efficacy may provide a necessary expectancy‐related foundation for teachers to adopt (usually quite demanding) mastery‐oriented student goals, emphasizing understanding, critical thinking and skill acquisition among their students. Besides these effect via student goals, such teachers may also see mastery‐oriented instruction (e.g. collaborative learning, formative assessments) as both feasible and effective, thus aligning their instructional design with their belief in students' potential for growth. Conversely, low self‐efficacy can be expected to limit a teacher's openness to implementing mastery‐oriented practices due to perceived risks or doubts about their ability to manage diverse student needs. Such cognitive constraints may instead lead to an overreliance on performance goals in students as well as performance‐oriented practices, where outcomes like standardized test scores are prioritized as clear markers of success.

### The present research

Building on previous work that linked teachers' personal achievement goals and self‐efficacy to instructional outcomes, our study advances the literature by integrating two traditionally separate strands of research—achievement goal and self‐efficacy approaches—while following up on the relevance of student‐oriented goals within the process translating teacher motivation into instructional behaviour. In addition to mastery and performance goals, we examine personal work‐avoidance goals as another personal goal predictor alongside the inclusion of self‐efficacy as a key expectancy‐related aspect of motivation, reflecting that teachers' beliefs in their abilities also influence their goal‐setting and classroom practices. Within such an integrated approach, considering student‐oriented goals seems particularly warranted as, besides more direct effects of personal motivations, student‐oriented goals could plausibly serve as an important process element that translates teachers' personal achievement goals and self‐efficacy into actionable instructional behaviours. Moreover, teachers' student‐oriented goals may represent a distinct construct in their own right, warranting their inclusion in theorizing on the processes linking teacher motivation to student outcomes.

In this study, we adopt a different approach from recent research to test these assumptions. While instructional practices are often assessed through teachers' or students' general reports of mastery‐ or performance‐based instruction, such methods may overlook the nuanced effects of different strategies and the motivations behind them. Therefore, a more detailed investigation is warranted to better understand the underlying processes (Daumiller et al., 2023). Unlike the broad reports typically used, our study employs a standardized lesson diary technique, which provides a fine‐grained view on instructional practices by repeatedly recording the practices applied in each lesson and is thus closer to the actual teaching situation. Using the standardized lesson diary, we explore diverse and specific aspects of teaching behaviour, building on the work of Daumiller et al. (2023).

## METHOD

To answer our research questions, we asked German secondary education teachers to fill out a baseline questionnaire about their teaching motivation (personal achievement goals, self‐efficacy and student‐oriented goals) and, over the course of five subsequent lessons, also standardized diaries concerning their specific teaching practices in these lessons. In part, this dataset was already used by Daumiller, Fasching, et al. (2022), with teachers' reports on their personal and student‐oriented goals for teaching. In the present work, we combine this data with novel data stemming from a lesson diary that the teachers filled out across five lessons after they made their assessments regarding the baseline questionnaire. The study was conducted in full accordance with the Ethical Guidelines of the German Association of Psychologists and the American Psychological Association, with written approval from the responsible Ministry of Education.

### Sample

We focused on teachers from general education secondary schools from lower tracks (called ‘Hauptschule’) in the southern part of Germany. We reached out to school principals by mail, providing them with written information about the study. This resulted in the participation of 37 schools (14.5% of those contacted). Within these schools, we invited 7th to 9th grade class teachers who taught mathematics to their main class. Teachers in these schools typically function as homeroom teachers, meaning they teach most subjects to one class rather than specializing in one or two subjects across multiple classes. Participation was voluntary and anonymous. We obtained informed consent from all participating teachers (for more details on the procedure, see Daumiller, Fasching, et al., 2022).

The final sample included 70 teachers, subsuming 42 women and 28 men, with a mean age of 43.7 years (*SD* = 10.6). The average teaching experience was 18 years (*SD* = 11.6). Through the lesson diary, a total of 345 assessments were collected regarding their specific teaching behaviours regarding each lesson. In addition to the sample of 70 teachers, 12 teachers signed up for the study but only completed the initial questionnaire and did not fill out the lesson diary. Therefore, their data were not included in the present investigation.

### Measures

All items were presented along with Likert‐type scales. We summarize all assessed scales, including example items and reliabilities (McDonald's ω), in Table 1. Teaching practices exhibited considerable between‐teacher variation but also substantial variance between different lessons of the same teacher (ICC1 = .35–.61, *p* < .05; see Table 1).

**TABLE 1:** Overview of the scales included in the baseline assessments and the lesson diary.

Scale	#	Potential range	ω	ICC1	Example item
Baseline					
Personal motivation					
Learning	9	1–5	.92	–	In my vocation, I aspire to improve my pedagogical knowledge and my competences
Performance approach	12	1–5	.97	–	In my vocation, I aspire my colleagues to realize that I teach better than other teachers
Performance avoidance	12	1–5	.96	–	In my vocation, I aspire my students not to believe I would master my job less sufficient than other teachers
Work avoidance	6	1–5	.82	–	I aspire to get through the day with little effort
Self‐efficacy	10	1–4	.81	–	I know that I can manage to teach even the most problematic students the material
Student‐oriented goals					
Mastery	8	1–6	.80	–	In my mathematics class, it is my main goal that my students continue to learn and improve themselves
Performance	12	1–6	.90	–	In my math class, my most important goal is that my students score well in examinations
Lesson Diary (Item stem: ‘In my lesson today in Mathematics …’)					
Specific teaching behaviours					
Interestingness	6	1–5	.81	.54	… I used examples from everyday life to show the students what mathematics can be used for
Cognitive stimulation	4	1–5	.71	.39	… I had my students explain their thought processes in detail
Individualization	3	1–5	.82	.50	… I varied the tasks to suit students of different abilities
Public negative feedback	3	1–5	.68	.61	… I told students in front of the whole class when they did badly
Autonomy support	6	1–5	.78	.51	… I encouraged the students to work independently
Structuring	2	1–5	.85	.38	… I emphasized the relationships between the topics covered
Collaboration	4	1–5	.91	.35	… I encouraged students to work together
Heterogeneous grouping	2	1–5	.80	.55	… the students worked together with bad and good students in each group
Homogeneous grouping	2	1–5	.75	.49	… the better students worked with each other and the worse students worked with each other
Competition	5	1–5	.76	.61	… I encouraged my students to compete with each other

*Note*: *#* denotes the number of items, ω the internal consistency of the respective scale, and ICC1 represents the proportion of variance at the level of the diaries that can be attributed to the individual teachers.

In the baseline questionnaire, we measured teachers' achievement goals with the Nitsche et al. (2011) questionnaire, distinguishing learning goals, performance approach goals, performance avoidance goals and work‐avoidance goals. Self‐efficacy beliefs for teaching were measured using a scale by Schmitz and Schwarzer (2002). Student‐oriented mastery goals and student‐oriented performance goals were measured with the Daumiller, Fasching, et al. (2022) scale.

In the subsequent lesson diaries, teachers reported on their specific teaching practices over the course of five subsequent lessons. The teachers were instructed to answer the diaries as soon as possible after the respective lesson, but in any case, on the same day. All lesson diary items were focused on the past lesson following the item stem ‘In my lesson today in mathematics …’. The specific teaching practices were measured analogously to Daumiller et al. (2023) and the established scales described therein, encompassing, based on Ames (1992) and Benning et al. (2019), 10 aspects that should be clearly assessable by teachers, containing both mastery (i.e. interestingness, cognitive stimulation, autonomy support, structuring, collaboration and heterogeneous grouping) as well as performance‐based aspects (i.e. public negative feedback, homogeneous grouping and competition).

### Analyses

We conducted two‐level path modelling with *Mplus 8.1* (Muthén & Muthén, 2017). In order to examine between‐teacher differences in their use of the distinct instructional practices across the 5 weeks, we focused on the teacher level (L2), wherein we regressed teachers' specific instructional practices on their (1) personal goals and self‐efficacy and (2) student‐oriented goals. We examined their direct associations as well as their joint effects within a total of three models. Missing values due to item non‐response occurred rarely (less than 4% per variable) and were handled through the FIML approach in *Mplus*.

As robustness checks, we conducted supplementary analyses in the form of 10 individual two‐level regression analyses. These were analogous to the comprehensive path model estimated in our main analysis, but instead of the multivariate approach, a single model was estimated for each of the teaching practices considered as dependent variables (see Data S1).

## RESULTS

Descriptively, the results (Table 2) indicated that teachers strongly endorsed learning goals and rather weakly pursued performance and work avoidance goals alongside average levels of self‐efficacy beliefs, with substantial differences between the different teachers (reflected in the standard deviations). Student‐oriented goals were rather strong for mastery goals and moderate for performance goals. Descriptively, there was rather small variability in self‐efficacy beliefs with rather positive expressions. Notably, we observed substantial differences between teachers regarding their instructional behaviours across lessons (reflected in large ICC1s), warranting the analysis of respective between‐teacher differences that were the focus of the present investigation.

**TABLE 2:** Descriptive statistics and bivariate correlations among the assessed constructs.

	Descriptive statistics					Bivariate correlations															
	*M*	*SD*	Min	Max	Skew	1	2	3	4	5	6	7	8	9	10	11	12	13	14	15	16
Personal motivation																					
[1] Learning	4.24	.52	2.11	5.00	−.87																
[2] Performance approach	1.81	.78	1.00	5.00	1.17	−.06															
[3] Performance avoidance	1.92	.74	1.00	3.42	.19	.01	.**27**														
[4] Work avoidance	1.79	.83	1.00	4.33	1.04	−.16	.**31**	.14													
[5] Self‐efficacy	3.09	.35	2.00	3.90	−.15	.**42**	−.16	−.18	−.20												
Student‐oriented goals																					
[6] Mastery	4.78	.47	3.80	5.80	−.07	.**22**	.06	−.09	−.05	.**26**											
[7] Performance	3.04	.61	1.32	4.55	−.18	.07	.**48**	.20	.21	−.03	.**29**										
Specific teaching behaviours (lesson diaries)																					
[8] Interestingness	2.95	.75	1.81	5.00	1.00	.08	.01	−.04	.01	.**47**	.**39**	.13									
[9] Cognitive stimulation	3.36	.69	1.45	5.00	.13	−.16	−.01	**−.24**	.10	.14	.**28**	−.02	.**35**								
[10] Individualization	3.07	1.00	1.00	5.00	−.09	.15	−.15	.04	−.02	.**23**	.15	.07	.**24**	.10							
[11] Public negative feedback	1.78	.68	1.00	5.00	1.87	**−.41**	.**24**	−.11	.21	**−.38**	−.10	.02	**−.12**	.02	−.05						
[12] Autonomy support	2.40	.93	1.00	5.00	1.02	−.10	**−.30**	.04	.02	.**31**	.17	−.05	.**20**	.**21**	.**61**	−.06					
[13] Structuring	3.53	.75	1.00	5.00	−.20	.07	−.03	−.16	.04	.**31**	.11	−.05	.**30**	.**29**	.**12**	−.10	.**16**				
[14] Collaboration	3.50	.69	2.10	4.83	.07	.06	−.36	**−.41**	−.09	.**30**	.20	−.15	.**20**	.**23**	.**24**	−.10	.**32**	.**20**			
[15] Heterogeneous grouping	3.60	.98	1.00	5.00	−.68	−.17	−.19	**−.44**	.18	−.04	.01	−.11	−.07	.**26**	.10	.**14**	.**17**	.08	.**52**		
[16] Homogeneous grouping	1.85	.69	1.00	3.25	.35	−.16	−.06	.06	−.05	−.17	−.10	.14	.03	−.05	.07	.01	.08	−.03	−.02	**−.28**	
[17] Competition	1.68	.82	1.00	5.00	2.90	−.19	−.10	−.04	.05	.13	.**22**	−.05	.**28**	.**14**	.06	.00	.**32**	.19	.11	−.02	.08

*Note*: *N* = 70 teachers with *N* = 345 standardized lesson diary entries regarding teaching behaviours. Statistically significant correlations are boldfaced. Correlations with personal motivation and student‐oriented goals are calculated on the teacher level with |*r*| > .21: *p* < .05, |*r*| > .30: *p* < .01, |*r*| > .40: *p* < .001; the other on the level of the diary entries with |*r*| > .10: *p* < .05, |*r*| > .14: *p* < .01, |*r*| > .19: *p* < .001.

The full results of our main analysis are presented in Table 3. The two‐level path modelling confirmed that personal performance goals were positively linked to student‐oriented performance goals: teachers who reported stronger personal performance goals than other teachers also tended to report stronger student‐oriented performance goals than other teachers. Unlike Daumiller, Fasching, et al. (2022) we did not observe a statistically significant linkage between personal learning goals and student‐oriented mastery goals; however, self‐efficacy beliefs were positively associated with the latter.

**TABLE 3:** Results of two‐level path modelling testing the linkages between personal motivation, student‐oriented goals and individual teaching practices.

	Interestingness	Cognitive stimulation	Individualization	Public neg. Feedback	Autonomy support	Structuring	Collaboration	Heterog. Grouping	Homog. Grouping	Competition	Student‐oriented mastery	Student‐oriented performance
Model 1: Personal motivation												
Learning	−.19 (.15)	−.29 (.16)	−.14 (.16)	−.31 (.12)	−.31 (.11)	−.08 (.14)	−.02 (.15)	−.12 (.15)	−.20 (.17)	−.31 (.13)		
Performance approach	.12 (.13)	.10 (.16)	−.20 (.12)	.25 (.14)	−.33 (.10)	.08 (.12)	−.30 (.13)	−.09 (.11)	−.17 (.18)	−.08 (.09)		
Performance avoidance	−.01 (.14)	−.27 (.13)	.07 (.11)	−.21 (.11)	.20 (.11)	−.18 (.14)	−.42 (.12)	−.51 (.11)	.13 (.16)	.03 (.10)		
Work avoidance	.11 (.10)	.16 (.13)	.08 (.13)	.09 (.12)	.18 (.12)	.13 (.11)	.10 (.12)	.25 (.15)	−.04 (.14)	.11 (.10)		
Self‐efficacy	.67 (.12)	.33 (.15)	.45 (.14)	−.21 (.12)	.48 (.13)	.43 (.17)	.31 (.16)	.03 (.15)	−.03 (.20)	.28 (.16)		
*R* ^2^	.34	.20	.21	.33	.32	.19	.47	.35	.08	.11		
Model 2: Student‐oriented goals												
Mastery	.45 (.12)	.37 (.14)	.30 (.14)	−.12 (.12)	.23 (.15)	.17 (.16)	.35 (.16)	.06 (.17)	−.09 (.20)	.27 (.14)		
Performance	.00 (.15)	−.15 (.15)	−.08 (.15)	.06 (.15)	−.12 (.14)	−.13 (.15)	−.29 (.16)	−.15 (.16)	.15 (.19)	−.13 (.13)		
*R* ^2^	.20	.12	.08	.01	.05	.03	.14	.02	.02	.07		
Model 3: Combined												
Personal motivation												
Learning	−.22 (.13)	−.31 (.15)	−.16 (.15)	−.31 (.12)	−.33 (.10)	−.08 (.15)	−.04 (.14)	−.13 (.15)	−.20 (.18)	−.33 (.12)	.08 (.13)	.07 (.11)
Performance approach	.07 (.12)	.10 (.17)	−.26 (.13)	.28 (.15)	−.37 (.12)	.13 (.12)	−.34 (.14)	−.12 (.12)	−.31 (.20)	−.07 (.09)	.14 (.10)	.44 (.09)
Performance avoidance	.01 (.12)	−.24 (.13)	.07 (.11)	−.21 (.11)	.21 (.11)	−.16 (.16)	−.41 (.13)	−.51 (.11)	.08 (.16)	.05 (.10)	−.05 (.12)	.09 (.09)
Work avoidance	.11 (.10)	.18 (.13)	.07 (.13)	.10 (.12)	.18 (.12)	.15 (.13)	.09 (.12)	.24 (.15)	−.09 (.15)	.12 (.10)	.01 (.11)	.13 (.11)
Self‐efficacy	.59 (.12)	.26 (.15)	.39 (.14)	−.20 (.13)	.44 (.12)	.42 (.17)	.26 (.15)	.00 (.15)	−.08 (.19)	.23 (.14)	.22 (.13)	.09 (.10)
Student‐oriented goals												
Mastery	.35 (.11)	.35 (.14)	.21 (.12)	−.01 (.13)	.17 (.12)	.08 (.16)	.23 (.13)	.02 (.15)	−.08 (.19)	.27 (.11)		
Performance	.00 (.13)	−.13 (.13)	.06 (.13)	−.05 (.12)	.03 (.14)	−.14 (.19)	.01 (.17)	.05 (.16)	.34 (.19)	−.10 (.15)		
*R* ^2^	.45	.30	.26	.33	.35	.20	.53	.35	.15	.17	.08	.29

The differences that emerged between the participating teachers in their specific teaching practices over the 5 weeks were explained to a substantial amount by the considered set of predictors, with partly different result patterns observed for the different instructional practices (*R* ^2^ = .15–.45). Specifically, student‐oriented mastery goals positively predicted interestingness, cognitive stimulation, individualization and collaboration, as expected and were also (but only in the combined model) linked to increased reports of competition. Besides these, student‐oriented performance goals were only slightly informative regarding these practices—they were statistically significantly linked only to increased homogeneous grouping. Regarding teachers' personal motivation, we found that learning goals were negatively linked to competition and public negative feedback; however, and contrary to our expectations, we did not find that learning goals were positively related to mastery teaching practices. Instead, they were even negatively linked to interestingness, autonomy support and cognitive stimulation. Personal performance approach goals went along with less individualization, more public negative feedback, less autonomy support and less use of collaboration, while personal performance avoidance goals went along with less collaboration and less heterogeneous grouping. Work avoidance goals were not statistically significantly linked to differences among the teachers in the considered teaching practices. Self‐efficacy, however, was a relatively influential predictor that was associated with increased interestingness, cognitive stimulation, individualization, autonomy support and structuring, but was not significantly linked to any of the performance‐based instructional practices.

Of note, while descriptively smaller, most of these linkages emerged in the combined model and when considering either personal motivation or student‐oriented goals as predictors alone, implying that student‐oriented goals were not a critical or full mediator of these linkages. Moreover, most of these effects also emerged in the individual regression analyses that we conducted as a form of a robustness test besides the comprehensive multivariate analysis in our main model and that are presented in the Table S1.

## DISCUSSION

Interested in the (cognitive) processes linking teachers' personal achievement goals and self‐efficacy with their instructional behaviours, we followed up on teachers' student‐oriented goals (Daumiller, Fasching, et al., 2022). In addition to personal goals, we extended prior research by also considering self‐efficacy as a critical expectancy‐related factor. We found evidence that especially student‐oriented mastery goals may be a relevant process element and require substantial levels of self‐efficacy to be facilitated, warranting a comprehensive consideration of goals *and* efficacy beliefs to better understand teachers goal pursuit in the classroom. Moreover, our findings highlighted the complex pathways through which teacher motivation manifests in specific instructional strategies. Through a standardized lesson diary approach, we were able to capture a fine‐grained view of teaching behaviours, finding evidence for different relationships that warrant a differentiated perspective for future research on this topic. Such a comprehensive perspective contributes to a deeper understanding of the cognitive processes involved in teaching and promises valuable insights for future research on teacher motivation (Lazarides et al., 2024).

Regarding the relationships with student‐oriented goals, our analysis revealed different relationships for teachers' personal goals and self‐efficacy. Self‐efficacy emerged as relevant predictor of student‐oriented mastery goals, underscoring the role of teachers' confidence in their abilities when setting goals aimed at fostering students' learning and development (Klassen et al., 2009; Tschannen‐Moran et al., 1998). This suggests that teachers who believe in their instructional efficacy are more likely to adopt goals that prioritize students' mastery and understanding. Conversely, personal learning goals, while positively associated with student‐oriented mastery goals in bivariate analyses, did not retain their significance when self‐efficacy was included in the multivariate model. This indicates that simply assuming similar value appraisals (e.g. ‘spilling over’ effects) is not sufficient to understand the effects of teacher motivation on their student‐oriented goals. For performance‐oriented student goals, the data showed that these were primarily predicted by teachers' own performance approach goals, whereas self‐efficacy did not play a significant role. This highlights the complexity of goal‐setting processes, suggesting that while mastery‐oriented goals are more expectancy‐dependent, performance‐oriented goals may be more closely tied to the teachers' own achievement goals and standards (Butler, 2007; Daumiller, Fasching, et al., 2022). These findings contribute to our understanding of the interplay between different types of goals and highlight the need to consider both personal and student‐oriented goals to more fully grasp the motivational dynamics at hand.

Further, our findings shed light on the role of self‐efficacy and personal goals in shaping instructional practices, and the relevance of student‐oriented goals for these processes. Self‐efficacy was positively associated with almost all considered mastery‐based practices, indicating that teachers with higher confidence in their teaching abilities are more likely to create classroom environments that emphasize understanding and skill development (Lüftenegger & Muth, 2024; Skaalvik & Skaalvik, 2023). This speaks to the role of teachers' self‐efficacy alongside their goals in facilitating mastery‐oriented practices (Bandura, 1997). This became especially clear when compared to learning goals: our findings implied that such at first sight favourable personal motivation does not directly lead a teacher using richer instructional practices. This makes sense as learning goals focus on teachers' own professional growth, resilience and continuous development rather than directly shaping instructional choices. In contrast, student‐oriented goals may offer a more immediate link to classroom behaviours, thus warranting their consideration when interested in the cognitive processes underlying more immediate motivated action of teachers in the classroom (Daumiller, Böheim, et al., 2025). Indeed, we found that student‐oriented mastery goals were positively associated with mastery‐based instructional strategies, supporting prior research on the link between mastery goal structures and student‐centered, cognitively engaging instruction (e.g. Ames, 1992; Khajavy et al., 2018; Skaalvik & Skaalvik, 2017).

Notably, however, also an unexpected finding emerged: student‐oriented mastery goals were also linked to an increased emphasis on competition. This might suggest that teachers could also perceive and implement such practices in a more constructive manner when pursuing strong mastery goals for their students—for example, by using peer performance as a benchmark to motivate student progress. In subjects such as mathematics, this might involve leveraging performance comparisons to encourage skill development while maintaining a supportive instructional climate. This finding echoes the complexity noted by Daumiller, Fasching, et al. (2022), where certain instructional strategies can serve dual purposes, fostering both performance and mastery goals depending on the context and implementation. This underscores the necessity of more specifically considering the context at hand (and what teachers think about it) when examining the relationships between teacher motivation and instructional behaviours.

Another notable finding is the lack of significant effects for work‐avoidance goals. While prior research has linked work‐avoidance goals to lower job engagement, reduced instructional quality and a reluctance to adopt innovative teaching methods (e.g. Nitsche et al., 2013; Retelsdorf et al., 2010), our findings suggest that these goals may not systematically translate into observable differences in daily teaching behaviours—at least not in the specific instructional practices assessed in this study. One possible explanation is that teachers with higher work‐avoidance tendencies might still adhere to institutional norms and baseline professional expectations, thereby masking potential negative effects within the lesson diaries. Alternatively, the impact of work‐avoidance goals may manifest in less directly observable ways, such as lower quality implementation of said teaching strategies, lower responsiveness to student needs, etc. Future research could explore these alternative pathways, perhaps by incorporating behavioural indicators of effort investment or examining the long‐term consequences of work‐avoidance motivation on teacher effectiveness and student outcomes.

The differential relations of our considered set of predictors with the specific instructional practices are another relevant finding of our study. While self‐efficacy and student‐oriented mastery goals were positively associated with most mastery‐oriented teaching behaviours, they were not linked to all considered practices. Notably, performance goals exhibited distinct and specific associations with teaching behaviours, underscoring the complexity of their role in instructional decision‐making. For instance, personal performance approach goals were linked to more public negative feedback, less autonomy support and reduced individualization and collaboration, confirming potential drawbacks of performance‐oriented motivations on one's teaching actions. While potential measurement issues cannot be entirely ruled out, these findings further support the notion that the translation of motivational orientations into concrete teaching behaviours depends on the specific instructional strategies being considered (Daumiller et al., 2023). For example, research on teacher autonomy support (Reeve, 2009; Ryan & Deci, 2017) suggests that autonomy‐supportive instructional strategies, which promote student engagement and intrinsic motivation, may be more readily adopted by teachers with high self‐efficacy and mastery‐oriented goals, whereas performance‐oriented teachers may be more inclined towards controlling strategies that emphasize external evaluation and compliance (Hornstra et al., 2015). These distinctions reinforce the argument that instructional decision‐making is shaped not only by broad motivational tendencies but also by teachers' perceptions of effectiveness, goal structures and classroom affordances (Lazarides & Schiefele, 2021). Rather than assuming a uniform relationship, future research should place greater emphasis on the cognitive processes underlying teachers' instructional choices—including their interpretations of success, risk assessments of different teaching strategies and beliefs about student learning potential. Future research should explore these dynamics using longitudinal designs alongside cognitive assessments (e.g. think‐aloud protocols) to capture the real‐time decision‐making processes underlying instruction.

Of note, even when student‐oriented goals were considered alongside personal motivation, most personal achievement goals and self‐efficacy beliefs remained statistically significant predictors of instructional practices—albeit with some descriptively weaker effects. In line with Daumiller, Fasching et al. (2022), this suggests that teachers' standards for their own competence and success extend beyond their explicit goals for students, mattering for their instructional behaviours. In other words, while student‐oriented goals provide a cognitive link between personal motivation and classroom practices, they likely do not fully mediate these relationships. Notably, these goals were assessed at the baseline alongside personal motivation, limiting mediation testing. Future research should refine its assessment to better capture their dynamic alignment with instructional decision‐making—ideally through state‐based measures such as experience sampling or integration within lesson diaries. Nevertheless, our findings highlight the need to consider additional process elements shaping how teachers' personal motivations translate into teaching behaviours. One promising candidate could be intended classroom goal structures (see Supporting Information S2)—the overarching learning environments teachers aim to create through their instructional choices (Daniels et al., 2013; Meece et al., 2006). While student‐oriented goals reflect teachers' aspirations for their (individual) students, intended classroom goal structures capture the broader motivational climate that teachers seek to establish.

These insights align with the broader discussion in this special issue, which seeks to illuminate key process elements in understanding the effects of teacher motivation. While our study highlights the relevance of student‐oriented goals alongside personal motivation and self‐efficacy, this perspective necessarily simplifies a more complex interplay of psychological factors. For example, beyond goals and efficacy, beliefs about the malleability of abilities (e.g. growth vs. fixed mindsets) might form a more general ‘meaning system’ that should underlie the formation of goals, self‐efficacy and subsequent instructional behaviours (Lüftenegger & Muth, 2024; Murphy et al., 2021). Moreover, the relationships among these constructs are likely to be bidirectional and dynamic, with personal goals and self‐efficacy not merely shaping instruction but also being shaped by the enacted structures and their outcomes. For instance, a teacher who successfully implements mastery‐oriented teaching practices and observes positive student responses may experience an increase in self‐efficacy, reinforcing their commitment to such goals and practices (in line with Tschannen‐Moran & Hoy, 2001). To further disentangle these dynamics, cognitive mapping approaches could be employed to pinpoint particularly influential leverage points for both theoretical understanding and practical intervention (especially on an ideographic level).

In sum, these findings underscore the need for a fine‐grained perspective on teaching behaviours, as simply distinguishing between mastery and performance practices seems insufficient to capture the full range of teachers' instructional practices and how they are driven by their goals. Future research should delve deeper into these dynamics, perhaps incorporating classroom observations or teacher interviews to better understand how exactly teachers' motivations translate into their daily teaching practices. Focusing on the construct of student‐oriented goals may help to think about and theoretically situate these processes.

### Limitations

Despite its strengths, our study also comes with several limitations that should be considered when interpreting the findings and that offer directions for future research. First, the reliance on teachers' self‐reports to measure instructional practices introduces the possibility of common method bias. Although we employed a standardized lesson diary to capture a fine‐grained view of teaching behaviours, self‐report measures can still be subject to social desirability and recall biases. Moreover, while using two different assessment methods, all measures were self‐reported by the teachers, thus hindering causal inferences beyond mere associations. Future studies could mitigate this concern by incorporating multiple data sources, such as student reports, classroom observations and video recordings, to triangulate data and obtain a more comprehensive view of instructional practices (Brophy & Good, 1986; Baumert & Kunter, 2013). Given established differences in teachers' and students' perceptions of instructional behaviour in the classroom (Praetorius et al., 2012), it can be assumed that if student reports had been used, the effects might have been smaller and potentially less differentiated across various instructional strategies. Consequently, the effects observed in our study should be cautiously interpreted as upper‐bound estimates of the relationships with teaching behaviour.

Second, our study focused on a specific educational context—secondary school mathematics teachers in lower‐track schools in southern Germany. This limits the generalizability of our findings to other subjects, educational levels and cultural contexts. For example, the goal‐setting and instructional practices of teachers in subjects such as languages or the arts might differ significantly from those in mathematics, which typically follows a more rigid curriculum that might provide fewer personal learning opportunities for teachers compared to other subjects that might offer more flexibility, where a teacher might—for instance—explore an unfamiliar novel with the students. Therefore, future research should replicate and extend our findings in diverse educational settings and subjects to examine the robustness and applicability of our results.

Third, the complexity of motivational processes and instructional practices warrants more sophisticated analytical techniques. Future research should explore the cognitive processes underlying teachers' goal‐setting and instructional decisions, for example by using interviews to gain deeper insights into their reasoning and decision‐making (Ericsson & Simon, 1993). Such qualitative approaches could also meaningfully complement quantitative findings and provide a richer understanding of the motivational dynamics at play. This is particularly relevant given that quantitative studies like the present one can only examine a predetermined and limited set of constructs. While we focused student‐oriented goals on the established mastery/performance goal dichotomy, it is of course also conceivable that further distinctions (e.g. performance approach and avoidance goals) and additional types of goals beyond competence‐related goals (e.g. intrinsic vs. extrinsic, see Jang, 2019; or focused on student well‐being or discipline) may also play an informative role.

## CONCLUSION

Our study emphasizes the relevance of student‐oriented goals alongside personal motivation to better understand the motivational underpinnings of effective instruction. The findings highlight that self‐efficacy matters alongside personal achievement goals for facilitating mastery‐oriented teaching, but also for shaping mastery goals set for their students. Through a standardized lesson diary approach, we provided a fine‐grained view of teaching behaviours, revealing nuanced relationships that extend beyond simple distinctions between mastery and performance. Our findings imply that the effects of teacher motivation on instructional practice are not straightforward in the sense of a ‘spilling over’ of desirable components of teacher motivation to teaching quality. Instead, cascades of specific cognitive drivers seem to underlie teaching behaviour embedded within larger goal‐meaning systems involving goals directed at teachers themselves as well as goals directed at their students.

## AUTHOR CONTRIBUTIONS

**Martin Daumiller:** Conceptualization; methodology; formal analysis; visualization; writing – review and editing; writing – original draft; investigation; software. **Hanna Gaspard:** Writing – review and editing. **Oliver Dickhäuser:** Conceptualization; methodology; funding acquisition; writing – review and editing. **Markus Dresel:** Conceptualization; methodology; resources; supervision; funding acquisition; validation.

## CONFLICT OF INTEREST STATEMENT

The authors declare no conflicts of interest.

## Supporting information


Data S1:

## Data Availability

The data that support the findings of this study are available on request from the corresponding author. The data are not publicly available due to privacy or ethical restrictions.

## References

[bjep12776-bib-0001] Ames, C. (1992). Classrooms: Goals, structures, and student motivation. Journal of Educational Psychology, 84(3), 261–271. 10.1037/0022-0663.84.3.261

[bjep12776-bib-0002] Bandura, A. (1986). Social foundations of thought and action: A social cognitive theory. Prentice‐Hall, Inc.

[bjep12776-bib-0003] Bandura, A. (1997). Self‐efficacy: The exercise of control. Freeman.

[bjep12776-bib-0004] Bardach, L. , & Klassen, R. M. (2021). Teacher motivation and student outcomes: Searching for the signal. Educational Psychologist, 56(4), 283–297. 10.1080/00461520.2021.1991799

[bjep12776-bib-0062] Baumert, J. , & Kunter, M. (2013). Stichwort: Professionelle Kompetenz von Lehrkräften. Stichwort: Zeitschrift für Erziehungswissenschaft, 277–337. 10.1007/s11618-006-0165-2

[bjep12776-bib-0005] Benning, K. , Praetorius, A.‐K. , Janke, S. , Dickhäuser, O. , & Dresel, M. (2019). Das Lernen als Ziel: Zur unterrichtlichen Umsetzung einer Lernzielstruktur [Learning as an objective: Instructional implementation of a mastery goal structure in classroom]. Unterrichtswissenschaft, 47, 523–545. 10.1007/s42010-019-00054-7

[bjep12776-bib-0061] Brophy, J. , & Good, T. (1986). Teacher behavior and student achievement. In M. C. Wittrock (Ed.), Handbook of research on teaching (3rd ed., pp. 328–375). MacMillan Reference Books.

[bjep12776-bib-0006] Butler, R. (2007). Teachers' achievement goal orientations and associations with teachers' help seeking: Examination of a novel approach to teacher motivation. Journal of Educational Psychology, 99(2), 241–252. 10.1037/0022-0663.99.2.241

[bjep12776-bib-0007] Butler, R. (2012). Striving to connect: Extending an achievement goal approach to teacher motivation to include relational goals for teaching. Journal of Educational Psychology, 104(3), 726–742. 10.1037/a0028613

[bjep12776-bib-0008] Butler, R. (2014). What teachers want to achieve and why it matters. In P. W. Richardson , S. A. Karabenick , & H. M. G. Watt (Eds.), Teacher motivation (pp. 20–35). Routledge. 10.4324/9780203119273-2

[bjep12776-bib-0009] Butler, R. , & Shibaz, L. (2008). Achievement goals for teaching as predictors of students' perceptions of instructional practices and students' help seeking and cheating. Learning and Instruction, 18(5), 453–467. 10.1016/j.learninstruc.2008.06.004

[bjep12776-bib-0010] Butler, R. , & Shibaz, L. (2014). Striving to connect and striving to learn: Influences of relational and mastery goals for teaching on teacher behaviors and student interest and help seeking. International Journal of Educational Research, 65, 41–53. 10.1016/j.ijer.2013.09.006

[bjep12776-bib-0011] Cho, Y. , & Shim, S. S. (2013). Predicting teachers' achievement goals for teaching: The role of perceived school goal structure and teachers' sense of efficacy. Teaching and Teacher Education, 32, 12–21. 10.1016/j.tate.2012.12.003

[bjep12776-bib-0012] Daniels, L. M. , Frenzel, A. C. , Stupnisky, R. H. , Stewart, T. L. , & Perry, R. P. (2013). Personal goals as predictors of intended classroom goals: Comparing elementary and secondary school pre‐service teachers. British Journal of Educational Psychology, 83(3), 396–413. 10.1111/j.2044-8279.2012.02069.x 23822528

[bjep12776-bib-0014] Daumiller, M. , Dickhäuser, O. , & Dresel, M. (2019). University instructors' achievement goals for teaching. Journal of Educational Psychology, 111(1), 131–148. 10.1037/edu0000271

[bjep12776-bib-0013] Daumiller, M. (2023). Achievement goals: The past, present, and possible future of achievement goal research in the context of learning and teaching. In G. Hagenauer , R. Lazarides , & H. Järvenoja (Eds.), Motivation and emotion in learning and teaching across educational contexts: Theoretical and methodological perspectives and empirical insights (pp. 35–53). Routledge. 10.4324/9781003303473-4

[bjep12776-bib-0300] Daumiller, M. , Böheim, R. , Alijagic, A. , Lewalter, D. , Gegenfurtner, A. , Seidel, T. , & Dresel, M. (2025). Guiding attention in the classroom: An eye‐tracking study on the associations between preservice teachers' goals and noticing of student interactions. British Journal of Educational Psychology, 95(Suppl. 1), S115–S132. 10.1111/bjep.12748 PMC12427153 40059539

[bjep12776-bib-0015] Daumiller, M. , & Dresel, M. (2023). Temporal dynamics between faculty goals, burnout/engagement, and performance in teaching and research: A latent change score approach. Contemporary Educational Psychology, 72, 102124. 10.1016/j.cedpsych.2022.102124

[bjep12776-bib-0017] Daumiller, M. , Fasching, M. S. , Dickhäuser, O. , & Dresel, M. (2023). Teachers' achievement goals and teaching practices: A standardized lesson diary approach. Teaching and Teacher Education, 127, 104079. 10.1016/j.tate.2023.104079

[bjep12776-bib-0016] Daumiller, M. , Fasching, M. , Steuer, G. , Dickhäuser, O. , & Dresel, M. (2022). From teachers' personal achievement goals to students' perceptions of classroom goal structures: Via student‐oriented goals and specific instructional practices. Teaching and Teacher Education, 111, 103617. 10.1016/j.tate.2021.103617

[bjep12776-bib-0018] Daumiller, M. , Janke, S. , Hein, J. , Rinas, R. , Dickhäuser, O. , & Dresel, M. (2021). Do teachers' achievement goals and self‐efficacy beliefs matter for students' learning experiences? Evidence from two studies on perceived teaching quality and emotional experiences. Learning and Instruction, 76, 101458. 10.1016/j.learninstruc.2021.101458

[bjep12776-bib-0019] Daumiller, M. , Janke, S. , Rinas, R. , Dickhäuser, O. , & Dresel, M. (2022). Need satisfaction and achievement goals of university faculty: An international study of their interplay and relevance. Higher Education, 83(6), 1183–1206. 10.1007/s10734-021-00736-1

[bjep12776-bib-0020] Elliot, A. J. , & Hulleman, C. S. (2017). Achievement goals. In A. J. Elliot , C. S. Dweck , & D. S. Yeager (Eds.), Handbook of competence and motivation: Theory and application (pp. 43–60). Guilford.

[bjep12776-bib-0064] Ericsson, K. A. , & Simon, H. A. (1993). Protocol analysis: Verbal reports as data (Rev. ed.). Bradford Books/MIT Press.

[bjep12776-bib-0021] Fives, H. , & Buehl, M. M. (2016). Teacher motivation: Self‐efficacy and goal orientation. In K. R. Wentzel & D. B. Miele (Eds.), Handbook of motivation at school: Second Edition (pp. 340–360). Taylor and Francis Inc. 10.4324/9781315773384

[bjep12776-bib-0022] Frenzel, A. C. , Goetz, T. , Lüdtke, O. , Pekrun, R. , & Sutton, R. E. (2009). Emotional transmission in the classroom: Exploring the relationship between teacher and student enjoyment. Journal of Educational Psychology, 101(3), 705–716. 10.1037/a0014695

[bjep12776-bib-0023] Gorozidis, G. , & Papaioannou, A. G. (2014). Teachers' motivation to participate in training and to implement innovations. Teaching and Teacher Education, 39, 1–11. 10.1016/j.tate.2013.12.001

[bjep12776-bib-0024] Han, J. , & Gao, C. (2023). Teachers' achievement goal orientations: A systematic review of 15 years of published empirical research. Teaching and Teacher Education, 128, 104146. 10.1016/j.tate.2023.104146

[bjep12776-bib-0059] Hornstra, L. , Mansfield, C. , Van der Veen, I. , Peetsma, T. , & Volman, M. (2015). Motivational teacher strategies: The role of beliefs and contextual factors. Learning Environments Research, 18, 363–392. 10.1007/s10984-015-9189-y

[bjep12776-bib-0065] Jang, H. R. (2019). Teachers' intrinsic vs. extrinsic instructional goals predict their classroom motivating styles. Learning and Instruction, 60, 286–300. 10.1016/j.learninstruc.2017.11.001

[bjep12776-bib-0026] Khajavy, G. H. , Bardach, L. , Hamedi, S. M. , & Lüftenegger, M. (2018). Broadening the nomological network of classroom goal structures using doubly latent multilevel modeling. Contemporary Educational Psychology, 52, 61–73. 10.1016/j.cedpsych.2017.10.004

[bjep12776-bib-0056] Klassen, R. M. , & Tze, V. M. (2014). Teachers' self‐efficacy, personality, and teaching effectiveness: A meta‐analysis. Educational Research Review, 12, 59–76. 10.1016/j.edurev.2014.06.001

[bjep12776-bib-0027] Klassen, R. M. , Bong, M. , Usher, E. L. , Chong, W. H. , Huan, V. S. , Wong, I. Y. , & Georgiou, T. (2009). Exploring the validity of a teachers' self‐efficacy scale in five countries. Contemporary Educational Psychology, 34(1), 67–76. 10.1016/j.cedpsych.2008.08.001

[bjep12776-bib-0028] Lauermann, F. , & Berger, J.‐L. (2021). Linking teacher self‐efficacy and responsibility with teachers' self‐reported and student‐reported motivating styles and student engagement. Learning and Instruction, 76, 101441. 10.1016/j.learninstruc.2020.101441

[bjep12776-bib-0029] Lauermann, F. , & Butler, R. (2021). The elusive links between teachers' teaching‐related emotions, motivations, and self‐regulation and students' educational outcomes. Educational Psychologist, 56(4), 243–249. 10.1080/00461520.2021.1991800

[bjep12776-bib-0031] Lazarides, R. , & Schiefele, U. (2021). Teacher motivation: Implications for instruction and learning. Introduction to the special issue. Learning and Instruction, 76, 101543. 10.1016/j.learninstruc.2021.101543

[bjep12776-bib-0033] Lazarides, R. , & Warner, L. M. (2020). Teacher self‐efficacy. In G. W. Noblit (Ed.), Oxford research encyclopedia of education (p. 1e22). Oxford University Press.

[bjep12776-bib-0030] Lazarides, R. , Buchholz, J. , & Rubach, C. (2018). Teacher enthusiasm and self‐efficacy, student‐perceived mastery goal orientation, and student motivation in mathematics classrooms. Teaching and Teacher Education, 69, 1–10.

[bjep12776-bib-0032] Lazarides, R. , Schiefele, U. , Daumiller, M. , & Dresel, M. (2024). From teacher motivation to instructional behavior: A systematic review of the mediating processes . https://osf.io/rgyat/

[bjep12776-bib-0034] Lüftenegger, M. , & Muth, J. (2024). Teachers' mindset meaning system: achievement goals, beliefs and classroom practices. Social Psychology of Education, 27(6), 1–20. 10.1007/s11218-024-09952-w

[bjep12776-bib-0060] Meece, J. L. , Anderman, E. M. , & Anderman, L. H. (2006). Classroom goal structure, student motivation, and academic achievement. Annual Review of Psychology, 57(1), 487–503. 10.1146/annurev.psych.56.091103.070258 16318604

[bjep12776-bib-0035] Murayama, K. , & Elliot, A. J. (2019). Achievement goals. In R. M. Ryan (Ed.), The Oxford handbook of human motivation (Vol. 2, pp. 227–245). Oxford University Press. 10.1093/oxfordhb/9780190666453.013.13

[bjep12776-bib-0036] Murphy, L. , Eduljee, N. B. , & Croteau, K. (2021). Teacher‐centered versus student‐centered teaching: Preferences and differences across academic majors. Journal of effective Teaching in Higher Education, 4(1), 18–39. 10.36021/jethe.v4i1.156

[bjep12776-bib-0037] Muthén, L. , & Muthén, B. (2017). *Mplus*. Version 8.1 [Computer Software].

[bjep12776-bib-0038] Nicholls, J. G. (1989). The competitive ethos and democratic education. Harvard University Press.

[bjep12776-bib-0039] Nitsche, S. , Dickhäuser, O. , Fasching, M. S. , & Dresel, M. (2011). Rethinking teachers' goal orientations: Conceptual and methodological enhancements. Learning and Instruction, 21(4), 574–586. 10.1016/j.learninstruc.2010.12.001

[bjep12776-bib-0040] Nitsche, S. , Dickhäuser, O. , Fasching, M. S. , & Dresel, M. (2013). Teachers' professional goal orientations: Importance for further training and sick leave. Learning and Individual Differences, 23, 272–278. 10.1016/j.lindif.2012.07.017

[bjep12776-bib-0041] Papaioannou, A. , & Christodoulidis, T. (2007). A measure of teachers' achievement goals. Educational Psychology, 27(3), 349–361. 10.1080/01443410601104148

[bjep12776-bib-0063] Praetorius, A. K. , Lenske, G. , & Helmke, A. (2012). Observer ratings of instructional quality: Do they fulfill what they promise? Learning and Instruction, 22(6), 387–400. 10.1016/j.learninstruc.2012.03.002

[bjep12776-bib-0057] Reeve, J. (2009). Why teachers adopt a controlling motivating style toward students and how they can become more autonomy supportive. Educational Psychologist, 44(3), 159–175. 10.1080/00461520903028990

[bjep12776-bib-0043] Retelsdorf, J. , & Günther, C. (2011). Achievement goals for teaching and teachers' reference norms: Relations with instructional practices. Teaching and Teacher Education, 27(7), 1111–1119. 10.1016/j.tate.2011.05.007

[bjep12776-bib-0042] Retelsdorf, J. , Butler, R. , Streblow, L. , & Schiefele, U. (2010). Teachers' goal orientations for teaching: Associations with instructional practices, interest in teaching, and burnout. Learning and Instruction, 20(1), 30–46. 10.1016/j.learninstruc.2009.01.001

[bjep12776-bib-0058] Ryan, R. M. , & Deci, E. L. (2017). Self‐determination theory: Basic psychological needs in motivation, development, and wellness. Guilford Publications.

[bjep12776-bib-0044] Schmitz, G. S. , & Schwarzer, R. (2002). Individuelle und kollektive Selbstwirksamkeitserwartung von Lehrern. In M. Jerusalem & D. Hopf (Eds.), Selbstwirksamkeit und motivationsprozesse in bildungsinstitutionen (pp. 192–214). Beltz.

[bjep12776-bib-0045] Schunk, D. H. , & DiBenedetto, M. K. (2020). Motivation and social cognitive theory. Contemporary Educational Psychology, 60, 101832. 10.1016/j.cedpsych.2019.101832

[bjep12776-bib-0046] Senko, C. (2016). Achievement goal theory: A story of early promises, eventual discords, and future possibilities. In K. R. Wentzel & D. B. Miele (Eds.), Handbook of motivation at school (Vol. 2, pp. 75–95). Routledge. 10.4324/9781315773384

[bjep12776-bib-0047] Skaalvik, E. M. , & Skaalvik, S. (2010). Teacher self‐efficacy and teacher burnout: A study of relations. Teaching and Teacher Education, 26(4), 1059–1069. 10.1016/j.tate.2009.11.001

[bjep12776-bib-0048] Skaalvik, E. M. , & Skaalvik, S. (2017). Motivated for teaching? Associations with school goal structure, teacher self‐efficacy, job satisfaction and emotional exhaustion. Teaching and Teacher Education, 67, 152–160. 10.1016/j.tate.2017.06.006

[bjep12776-bib-0049] Skaalvik, E. M. , & Skaalvik, S. (2023). Collective teacher culture and school goal structure: Associations with teacher self‐efficacy and engagement. Social Psychology of Education, 26(4), 945–969. 10.1007/s11218-023-09778-y

[bjep12776-bib-0050] Tschannen‐Moran, M. , & Hoy, A. W. (2001). Teacher efficacy: Capturing an elusive construct. Teaching and Teacher Education, 17(7), 783–805. 10.1016/s0742-051x(01)00036-1

[bjep12776-bib-0051] Tschannen‐Moran, M. , Hoy, A. W. , & Hoy, W. K. (1998). Teacher efficacy: Its meaning and measure. Review of Educational Research, 68(2), 202–248. 10.3102/00346543068002202

[bjep12776-bib-0052] Urdan, T. , & Kaplan, A. (2020). The origins, evolution, and future directions of achievement goal theory. Contemporary Educational Psychology, 61, 101862. 10.1016/j.cedpsych.2020.101862

[bjep12776-bib-0053] Watt, H. M. , Butler, R. , & Richardson, P. W. (2021). Antecedents and consequences of teachers' goal profiles in Australia and Israel. Learning and Instruction, 76, 101491. 10.1016/j.learninstruc.2021.101491

[bjep12776-bib-0054] Wolters, C. A. , & Daugherty, S. G. (2007). Goal structures and teachers' sense of efficacy: Their relation and association to teaching experience and academic level. Journal of Educational Psychology, 99(1), 181–193. 10.1037/0022-0663.99.1.181

[bjep12776-bib-0055] Zee, M. , & Koomen, H. M. (2016). Teacher self‐efficacy and its effects on classroom processes, student academic adjustment, and teacher well‐being: A synthesis of 40 years of research. Review of Educational Research, 86(4), 981–1015. 10.3102/0034654315626801

