# Supplementary material for: Bridging teacher motivation and instruction: Relevance of student‐oriented goals for teaching alongside personal achievement goals and self‐efficacy
Source: Br J Educ Psychol. 2025 Apr 17;95(Suppl 1):S98–S114. doi: 10.1111/bjep.12776 (PMC12427154; doi:10.1111/bjep.12776)
Supplement: Supplementary file 1 — Data S1: [file BJEP-95-S98-s001.docx]

**Bridging Teacher Motivation and Instruction: How Student-Oriented Goals Shape Teaching Alongside Teachers’ Personal Achievement Goals and Self-Efficacy**

**- Supplemental Information -**

This document contains materials designed to supplement the main text. The materials include the following:

1. Table S1: Results of the Ten Individual Two-Level Regression Analyses.
2. Additional Text and Analyses on Classroom-Goal structures

Table S1

*Results of the Ten Individual Two-Level Regression Analyses*

|  | Interesting- ness | Cognitive stimulation | Individual- ization | Public neg. feedback | Autonomy support | Structuring | Colla- boration | Heterog. Grouping | Homog. Grouping | Competition |
| --- | --- | --- | --- | --- | --- | --- | --- | --- | --- | --- |
| Personal motivation |  |  |  |  |  |  |  |  |  |  |
| Learning | **-.22 (.12)** | **-.30 (.15)** | .01 (.16) | **-.31 (.12)** | **-.32 (.11)** | -.07 (.15) | -.04 (.14) | -.18 (.14) | -.10 (.17) | **-.33 (.12)** |
| Performance approach | .08 (.13) | .11 (.17) | **-.28 (.15)** | **.29 (.15)** | **-.39 (.12)** | .14 (.13) | **-.34 (.14)** | -.12 (.13) | -.30 (.21) | -.07 (.09) |
| Performance avoidance | .01 (.12) | **-.25 (.12)** | .09 (.14) | -.21 (.10) | .22 (.12) | -.17 (.16) | **-.42 (.13)** | **-.50 (.10)** | .08 (.16) | .05 (.10) |
| Work avoidance | .11 (.10) | .18 (.13) | .06 (.14) | .10 (.12) | .18 (.13) | .15 (.13) | .09 (.12) | .26 (.16) | -.12 (.15) | .12 (.10) |
| Self-efficacy | **.59 (.13)** | **.26 (.14)** | .23 (.15) | -.20 (.13) | **.43 (.13)** | **.41 (.17)** | .25 (.16) | .08 (.14) | -.22 (.15) | .23 (.14) |
| Student-oriented goals |  |  |  |  |  |  |  |  |  |  |
| Mastery | **.35 (.11)** | **.35 (.14)** | .10 (.13) | -.01 (.13) | .17 (.13) | .07 (.16) | **.23 (.13)** | .06 (.12) | -.15 (.15) | **.27 (.11)** |
| Performance | .02 (.13) | -.13 (.13) | .14 (.13) | -.05 (.12) | .04 (.15) | -.13 (.20) | .02 (.17) | .03 (.15) | **.36 (.16)** | -.10 (.15) |
| *R***²** | .46 | .30 | .15 | .33 | .35 | .19 | .53 | .37 | .18 | .17 |

*Note*. Presented are standardized regression weights with their standard errors in brackets. Statistically significant coefficients are boldfaced. Effects that were statistically significant in the multivariate model (see Table 3) are highlighted with gray background.

**S2. Text and Additional Analyses on Intended Classroom-Goal structures**

In our dataset, we also have a measure of intended classroom goal structures. However, we are highly cautious in interpreting these data due to several substantial limitations. First, this construct was measured retrospectively, alongside teaching practices in the lesson diary, but after the teaching had already occurred. As such, teachers’ reports may have been influenced by their reflections on the lessons rather than representing a separate, pre-existing motivational construct. Second, the measure consists of only a few items, which might not adequately capture the full complexity of intended classroom goal structures. Nonetheless, for transparency, we present these findings while emphasizing the need for careful interpretation.

Figure S2.1. Differentiation of teachers’ student-directed goals in the theoretical model by Daumiller et al. (2022).

The rationale for additionally including intended classroom goal structures as another process element alongside student-oriented goals (see Figure S2.1) was grounded in prior research suggesting that these structures represent a key process element between teacher motivation and instructional behaviors. In fact, similar to our rationale for student-oriented goals, Daniels et al. (2013) provided a similar argument by investigating intended classroom goal structures in terms of the overall goal structures teachers intend for their classroom environment in relation to teachers’ personal achievement goals. As argued in the discussion of the present article, both student-oriented goals and intended classroom goal structures may be considered as mediators between teachers’ personal motivation and their instructional behaviors. To this end, the category of student-directed goals in the theoretical model by Daumiller et al. (2022) could be expanded by distinguishing between student-oriented goals in a narrower sense and intended classroom goal structures (Figure 1).

Intended classroom goal structures reflect the broader learning environment that teachers aim to create and have been proposed to stem from both teachers’ personal achievement goals and self-efficacy beliefs. Prior studies (e.g., Daniels et al., 2013) indicate that mastery-oriented classroom structures can foster student engagement and deep learning, whereas performance-oriented structures may emphasize competition and social comparison. In additional analyses, we therefore additionally explored the inclusion of intended classroom goal structures as a meaningful pathway linking teacher motivation to instructional practices, alongside student-oriented goals. Analogously to Daniels et al. (2013), intended classroom goal structures were measured in the teaching diary with a German version of the established and widely used PALS scales (Midgley et al., 2000; Retelsdorf et al., 2010), assessing intended mastery practices and intended performance practices (see Table S2.1).

Table S2.1

| Scale | # | Potential range | ω | ICC1 | Example item |
| --- | --- | --- | --- | --- | --- |
| **Lesson Diary** (Item stem: In my lesson today in Mathematics ...) | | | | | |
| **Intended classroom goal structure** | | | | | |
| Mastery | 3 | 1–6 | .81 | .51 | … I made a special effort to recognize the individual progress of my students, even if they were in the lower performance range. |
| Performance | 2 | 1–6 | .75 | .77 | … I pointed out the good students as role models for the others. |

Descriptively (see Table S2.2), the results indicated that teachers reported relatively strong endorsement of mastery-oriented classroom goal structures and moderate endorsement of performance-oriented structures. Two-level path modeling (Table S2.3) pointed to intended mastery goal structures were positively predicted by self-efficacy beliefs and student-oriented mastery goals, and negatively associated with performance-avoidance goals. Intended performance goal structures, however, were only negatively linked to teachers’ personal learning goals. Regarding teaching behaviors, intended mastery goal structures were positively associated with several mastery-based instructional practices, including interestingness and autonomy support. Somewhat surprisingly, intended performance goal structures were also positively related to individualization and structuring, which are typically considered mastery-oriented practices. This suggests that performance-oriented structures may not always be detrimental but could, in some cases, support structured and individualized learning environments. Additionally, public negative feedback was reported less frequently by teachers who endorsed mastery goal structures.

Given the mentioned limitations, the results should be interpreted with caution. However, they also highlight the potential value of considering intended classroom goal structures alongside student-oriented goals in future research on the cognitive pathways linking teacher motivation and instructional behavior.

**References**

Daniels, L. M., Frenzel, A. C., Stupnisky, R. H., Stewart, T. L., & Perry, R. P. (2013). Personal goals as predictors of intended classroom goals: Comparing elementary and secondary school pre‐service teachers. *British Journal of Educational Psychology*, *83*(3), 396-413. <https://doi.org/10.1111/j.2044-8279.2012.02069.x>

Daumiller, M., Fasching, M., Steuer, G., Dickhäuser, O., & Dresel, M. (2022). From teachers’ personal achievement goals to students’ perceptions of classroom goal structures: Via student-oriented goals and specific instructional practices. *Teaching and Teacher Education*, *111*, Article 103617. <https://doi.org/10.1016/j.tate.2021.103617>

Midgley, C., Maehr, M. L., Hruda, L. Z., Anderman, E., Anderman, L., Freeman, K. E., & Urdan, T. (2000). Manual for the patterns of adaptive learning scales. *Ann Arbor: University of Michigan*, 734-763.

Retelsdorf, J., Butler, R., Streblow, L., & Schiefele, U. (2010). Teachers' goal orientations for teaching: Associations with instructional practices, interest in teaching, and burnout. *Learning and Instruction*, *20*(1), 30-46. <https://doi.org/10.1016/j.learninstruc.2009.01.001>

Table S2.2

*Descriptive Statistics and Bivariate Correlations Among the Assessed Constructs Including Indented Goal Structures*

|  | Descriptive statistics | | | | |  | Bivariate correlations | | | | | | | | | | | | | | | | | |  |
| --- | --- | --- | --- | --- | --- | --- | --- | --- | --- | --- | --- | --- | --- | --- | --- | --- | --- | --- | --- | --- | --- | --- | --- | --- | --- |
|  | *M* | *SD* | Min | Max | Skew |  | 1 | 2 | 3 | 4 | 5 | 6 | 7 | 8 | 9 | 10 | 11 | 12 | 13 | 14 | 15 | 16 | 17 | 18 | |
| **Personal motivation** |  |  |  |  |  |  |  |  |  |  |  |  |  |  |  |  |  |  |  |  |  |  |  |  | |
| [1] Learning | 4.24 | 0.52 | 2.11 | 5.00 | -0.87 |  |  |  |  |  |  |  |  |  |  |  |  |  |  |  |  |  |  |  | |
| [2] Performance approach | 1.81 | 0.78 | 1.00 | 5.00 | 1.17 |  | –.06 |  |  |  |  |  |  |  |  |  |  |  |  |  |  |  |  |  | |
| [3] Performance avoidance | 1.92 | 0.74 | 1.00 | 3.42 | 0.19 |  | .01 | **.27** |  |  |  |  |  |  |  |  |  |  |  |  |  |  |  |  | |
| [4] Work avoidance | 1.79 | 0.83 | 1.00 | 4.33 | 1.04 |  | –.16 | **.31** | .14 |  |  |  |  |  |  |  |  |  |  |  |  |  |  |  | |
| [5] Self-efficacy | 3.09 | 0.35 | 2.00 | 3.90 | -0.15 |  | **.42** | –.16 | –.18 | –.20 |  |  |  |  |  |  |  |  |  |  |  |  |  |  | |
| **Student-oriented goals** |  |  |  |  |  |  |  |  |  |  |  |  |  |  |  |  |  |  |  |  |  |  |  |  | |
| [6] Mastery | 4.78 | 0.47 | 3.80 | 5.80 | -0.07 |  | **.22** | .06 | –.09 | –.05 | **.26** |  |  |  |  |  |  |  |  |  |  |  |  |  | |
| [7] Performance | 3.04 | 0.61 | 1.32 | 4.55 | -0.18 |  | .07 | **.48** | .20 | .21 | –.03 | **.29** |  |  |  |  |  |  |  |  |  |  |  |  | |
| **Intended goal structure** |  |  |  |  |  |  |  |  |  |  |  |  |  |  |  |  |  |  |  |  |  |  |  |  | |
| [8] Mastery structure | 4.21 | 0.83 | 1.75 | 6.00 | -0.07 |  | **.25** | **–.27** | **–.35** | **–.23** | **.48** | .20 | –.08 |  |  |  |  |  |  |  |  |  |  |  | |
| [9] Performance structure | 2.00 | 1.22 | 1.00 | 6.00 | 1.89 |  | –.08 | –.11 | –.09 | –.01 | .21 | **.28** | –.01 | .29 |  |  |  |  |  |  |  |  |  |  | |
| **Specific teaching behaviors** |  |  |  |  |  |  |  |  |  |  |  |  |  |  |  |  |  |  |  |  |  |  |  |  | |
| [10] Interestingness | 2.95 | 0.75 | 1.81 | 5.00 | 1.00 |  | .08 | .01 | –.04 | .01 | **.47** | **.39** | .13 | **.28** | **.33** |  |  |  |  |  |  |  |  |  | |
| [11] Cognitive stimulation | 3.36 | 0.69 | 1.45 | 5.00 | 0.13 |  | –.16 | –.01 | **–.24** | .10 | .14 | **.28** | –.02 | **.22** | **.26** | **.35** |  |  |  |  |  |  |  |  | |
| [12] Individualization | 3.07 | 1.00 | 1.00 | 5.00 | -0.09 |  | .15 | –.15 | .04 | –.02 | **.23** | .15 | .07 | **.33** | **.29** | **.24** | .10 |  |  |  |  |  |  |  | |
| [13] Public negative feedback | 1.78 | 0.68 | 1.00 | 5.00 | 1.87 |  | **–.41** | **.24** | –.11 | .21 | **–.38** | –.10 | .02 | **–.20** | **.13** | **–.12** | .02 | –.05 |  |  |  |  |  |  | |
| [14] Autonomy support | 2.40 | 0.93 | 1.00 | 5.00 | 1.02 |  | –.10 | **–.30** | .04 | .02 | **.31** | .17 | –.05 | **.35** | **.42** | **.20** | **.21** | **.61** | –.06 |  |  |  |  |  | |
| [15] Structuring | 3.53 | 0.75 | 1.00 | 5.00 | -0.20 |  | .07 | –.03 | –.16 | .04 | **.31** | .11 | –.05 | **.26** | **.22** | **.30** | **.29** | **.12** | –.10 | **.16** |  |  |  |  | |
| [16] Collaboration | 3.50 | 0.69 | 2.10 | 4.83 | 0.07 |  | .06 | –.36 | **–.41** | –.09 | **.30** | .20 | –.15 | **.27** | **.17** | **.20** | **.23** | **.24** | –.10 | **.32** | **.20** |  |  |  | |
| [17] Heterogeneous grouping | 3.60 | 0.98 | 1.00 | 5.00 | -0.68 |  | –.17 | –.19 | **–.44** | .18 | –.04 | .01 | –.11 | –.03 | .08 | –.07 | **.26** | .10 | **.14** | **.17** | .08 | **.52** |  |  | |
| [18] Homogeneous grouping | 1.85 | 0.69 | 1.00 | 3.25 | 0.35 |  | –.16 | –.06 | .06 | –.05 | –.17 | –.10 | .14 | –.02 | .11 | .03 | –.05 | .07 | .01 | .08 | –.03 | –.02 | **–.28** |  | |
| [19] Competition | 1.68 | 0.82 | 1.00 | 5.00 | 2.90 |  | –.19 | –.10 | –.04 | .05 | .13 | **.22** | –.05 | **.20** | **.54** | **.28** | **.14** | .06 | .00 | **.32** | .19 | .11 | –.02 | .08 | |

*Note*. *N* = 70 teachers with *N* = 345 standardized lesson diary entries regarding intended goal structures and teaching behaviors. Statistically significant correlations are boldfaced. Correlations with personal motivation and student-oriented goals are calculated on the teacher level with |*r*|>.21: *p* <.05, |*r*|>.30: *p* <.01, |*r*|>.40: *p* <.001, the other on the level of the diary entries with |*r*|>.10: *p* <.05, |*r*|>.14: *p* <.01, |*r*|>.19: *p* <.001.

Table S2.3

*Results of the Structural Equation Model Testing the Pathways Summarized in Figure 1*

| **Associations between personal motivation, student-oriented goals, and intended goal structures** | | | | | | | | | | | | |  |
| --- | --- | --- | --- | --- | --- | --- | --- | --- | --- | --- | --- | --- | --- |
|  | Student-oriented mastery | | | Student-oriented performance | | | Intended Mastery Structure | | | Intended Performance Structure | | |  |
| **Personal motivation** |  | | |  | | |  | | |  | | |  |
| Learning | .08 (.13) | | | -.05 (.11) | | | .09 (.14) | | | **-.26 (.12)** | | |  |
| Performance approach | .14 (.10) | | | **.40 (.10)** | | | -.12 (.10) | | | -.08 (.12) | | |  |
| Performance avoidance | -.05 (.12) | | | .05 (.11) | | | **-.30 (.11)** | | | -.01 (.11) | | |  |
| Work avoidance | .01 (.11) | | | .15 (.13) | | | -.10 (.11) | | | .06 (.13) | | |  |
| Self-efficacy | **.22 (.13)** | | | .05 (.11) | | | **.41 (.13)** | | | .25 (.14) | | |  |
| **Student-oriented goals** |  | | |  | | |  | | |  | | |  |
| Mastery |  | | |  | | | **.30 (.10)** | | | .10 (.12) | | |  |
| Performance |  | | |  | | | -.04 (.15) | | | .02 (.12) | | |  |
| *R*² | *.08 (.05)* | | | *.22 (.09)* | | | *.43 (.09)* | | | *.17 (.11)* | | |  |
| **Between-subject associations of personal motivation, student-oriented goals, and intended goal structures with individual teaching practices** | | | | | | | | | | | | | |
|  | Interesting- ness | Cognitive stimulation | Individual- ization | | Public neg. feedback | Autonomy support | Structuring | Colla- boration | Heterog. Grouping | | Homog. Grouping | Competition | |
| **Personal motivation** |  |  |  | |  |  |  |  |  | |  |  | |
| Learning | **-.26 (.12)** | -.25 (.16) | -.03 (.12) | | -.18 (.12) | **-.23 (.08)** | -.02 (.14) | -.06 (.15) | -.12 (.14) | | -.11 (.16) | **-.22 (.10)** | |
| Performance approach | .16 (.11) | .14 (.17) | -.18 (.10) | | **.25 (.15)** | **-.30 (.10)** | .20 (.12) | **-.31 (.15)** | -.15 (.14) | | -.27 (.19) | .03 (.09) | |
| Performance avoidance | .18 (.10) | -.18 (.14) | .15 (.10) | | **.32 (.11)** | **.32 (.10)** | -.05 (.14) | **-.37 (.14)** | **-.62 (.13)** | | .12 (.16) | **.20 (.09)** | |
| Work avoidance | .16 (.08) | .18 (.12) | .08 (.08) | | .04 (.13) | **.18 (.09)** | .16 (.13) | .11 (.11) | .23 (.15) | | -.10 (.15) | .12 (.09) | |
| Self-efficacy | **.26 (.12)** | .14 (.15) | .16 (.11) | | -.11 (.15) | .18 (.12) | .20 (.16) | .16 (.17) | .08 (.18) | | -.12 (.21) | -.10 (.12) | |
| **Student-oriented goals** |  |  |  | |  |  |  |  |  | |  |  | |
| Mastery | **.22 (.10)** | **.27 (.13)** | .03 (.10) | | -.06 (.13) | .00 (.10) | -.05 (.13) | .20 (.14) | .04 (.13) | | -.17 (.16) | .06 (.09) | |
| Performance | **-.26 (.11)** | -.01 (.14) | .09 (.11) | | .07 (.14) | .05 (.10) | -.08 (.14) | -.10 (.15) | -.09 (.19) | | .36 (.19) | -.02 (.10) | |
| **Intended goal structure** |  |  |  | |  |  |  |  |  | |  |  | |
| Mastery | **.60 (.13)** | .17 (.20) | .24 (.14) | | **-.41 (.22)** | **.34 (.17)** | .35 (.19) | .18 (.24) | -.25 (.24) | | .04 (.28) | **.46 (.14)** | |
| Performance | **.28 (.09)** | .22 (.16) | **.57 (.09)** | | .29 (.18) | **.48 (.11)** | **.33 (.11)** | .08 (.15) | .02 (.24) | | .32 (.19) | **.58 (.10)** | |
| *R*² | *.86 (.10)* | *.38 (.12)* | *.65 (.13)* | | *.42 (.19)* | *.70 (.10)* | *.42 (.13)* | *.57 (.13)* | *.42 (.13)* | | *.25 (.11)* | *.73 (.11)* | |

Continued on next page.

Table S2.3 continued.

| **Within-subject associations of intended goal structures with individual teaching practices** | | | | | | | | | | |  |
| --- | --- | --- | --- | --- | --- | --- | --- | --- | --- | --- | --- |
|  | Interesting- ness | Cognitive stimulation | Individual- ization | Public neg. feedback | Autonomy support | Structuring | Colla- boration | Heterog. Grouping | Homog. Grouping | Competition | |
| **Intended goal structure** |  |  |  |  |  |  |  |  |  |  | |
| Mastery | -.02 (.07) | .16 (.08) | **.33 (.07)** | -.05 (.09) | **.25 (.07)** | .12 (.07) | .13 (.11) | .09 (.09) | -.01 (.09) | -.13 (.11) | |
| Performance | .05 (.07) | **.13 (.05)** | **-.11 (.05)** | .05 (.07) | .07 (.06) | -.01 (.07) | .09 (.07) | .20 (.07) | .08 (.09) | .11 (.06) | |
| *R*² | *.00 (.01)* | *.05 (.03)* | *.11 (.04)* | *.00 (.01)* | *.07 (.04)* | *.02 (.02)* | *.03 (.03)* | *.05 (.03)* | *.01 (.01)* | *.02 (.03)* | |

*Note*. Presented are standardized regression weights with their standard errors in brackets. Statistically significant coefficients are boldfaced. Direct effects are highlighted with gray background, alongside the longer side paths (curvy lines in Figure S2.1).
